# Ectopic otoconial formation in the lagena of the pigeon inner ear

**DOI:** 10.1242/bio.034462

**Published:** 2018-06-26

**Authors:** E. Pascal Malkemper, Matthew J. Mason, Daniel Kagerbauer, Simon Nimpf, David A. Keays

**Affiliations:** 1 Research Institute of Molecular Pathology (IMP), Vienna Biocenter (VBC), Campus Vienna Biocenter 1, Vienna 1030, Austria; 2 University of Cambridge, Department of Physiology, Development & Neuroscience, Downing Street, Cambridge CB2 3EG, UK; 3 TU Wien, Atominstitut, Stadionallee 2, 1020 Vienna, Austria

**Keywords:** Inner ear, Otolith, Vestibular organs, Avian lagena, Tegmentum vasculosum, Dark cells, Magnetoreception

## Abstract

The vertebrate inner ear contains vestibular receptors with dense crystals of calcium carbonate, the otoconia. The production and maintenance of otoconia is a delicate process, the perturbation of which can lead to severe vestibular dysfunction in humans. The details of these processes are not well understood. Here, we report the discovery of a new otoconial mass in the lagena of adult pigeons that was present in more than 70% of birds. Based on histological, tomographic and elemental analyses, we conclude that the structure likely represents an ectopically-formed otoconial assembly. Given its frequent natural occurrence, we suggest that the pigeon lagena is a valuable model system for investigating misregulated otoconial formation.

This article has an associated First Person interview with the first author of the paper.

## Abstract (precis)

**Summary:** We identified a new assembly of otoconia in the pigeon lagena. The structure is not innervated or associated with hair cells. Our finding provides new insights into ectopic otoconial formation.

## INTRODUCTION

The inner ear of vertebrates contains several sensory epithelia that enable the detection of sound, acceleration and gravity. In birds, the basilar papilla (equivalent to the mammalian organ of Corti) is responsible for hearing, the semicircular canals perceive rotational acceleration, and the sacculus and utriculus detect linear acceleration (Manley, 1990). The latter two organs, together with an additional inner ear organ called the lagena, contain dense aggregates of proteins and calcium carbonate (CaCO_3_) crystals called otoconia. Otoconia are formed during embryonic development and maintained during adulthood (Ross, 1979; Thalmann et al., 2001). They are embedded within a gelatinous matrix, the otolithic membrane, lying above sensory hair cells. Relative movement of these otoconia result in the application of force on the stereocilia of the underlying hair cells, facilitating mechanotransduction.

The lagena is found in birds, fish, amphibians, reptiles and monotremes but not therian mammals; its function is incompletely understood (reviewed in Khorevin, 2008). In fish it is functionally and anatomically associated with the sacculus, fulfilling a primarily auditory, but also vestibular, function (Popper and Fay, 1999). In amphibians, the lagena has a vestibular role and is also sensitive to low-frequency seismic vibrations (Lewis et al., 1982). In birds and reptiles, the lagena represents a separate endorgan at the apical tip of the auditory basilar papilla (Smith and Takasaka, 1971). The structural similarity to the vestibular utricle and saccule, the absence of responses to auditory stimuli, and the exclusive innervation of vestibular brainstem nuclei render a vestibular function of the avian lagena likely (Kaiser and Manley, 1996; Manley et al., 1991). The striola of the lagena is aligned at an angle of 31-45° to the perpendicularly-aligned utricle and saccule and thus would collect useful additional information in three-dimensional space (Ishiyama, 1995). Additionally, synchrotron x-ray fluorescence (XRF) measurements have revealed iron in the otoconial layer of the lagena, which has led investigators to hypothesise that it could be involved in magnetic orientation (Harada, 2002, 2008; Harada et al., 2001; Wu and Dickman, 2011).

The correct formation, anchoring and maintenance of vestibular otoconia is essential for the sense of balance. Otoconia are formed during embryonic development by a series of temporally- and spatially-coordinated cellular and extracellular events (reviewed in Hughes et al., 2006; Lundberg et al., 2015). First, an organic matrix, consisting of mucoproteins and mucopolysaccharides, is produced by cells of non-sensory epithelia. Next, calcium (Ca^2+^) and bicarbonate ions (HCO_3_^−^), are concentrated within the endolymph, enabling the formation of crystalline seeds. These crystals increase in size, fuse in an organized pattern and are then anchored to sensory hair cells via the gelatinous matrix. Otoconia are renewed throughout life, but turnover rates are very low (Ross, 1979). The lack of otoconia, crystallization at undesired positions and otoconial displacement all have disastrous consequences for vestibular function: otoconial disorders collectively represent one of the major aging-associated diseases in humans (Agrawal et al., 2009; Lundberg et al., 2015). The intricate mechanisms governing correct otoconial assembly and maintenance are still not sufficiently understood, although animal models with otoconial defects, predominantly mice and bony fish, have been helpful in understanding the molecular details of vestibular organ development (Hughes et al., 2006).

Here, we report the discovery of a novel otoconial mass located at the junction between the lagena and basilar papilla in adult pigeons. Based on its composition and structure we conclude that these otoconia are ectopically-formed, and will be valuable for investigating misregulated otoconial formation.

## RESULTS

**Fig. 1.**
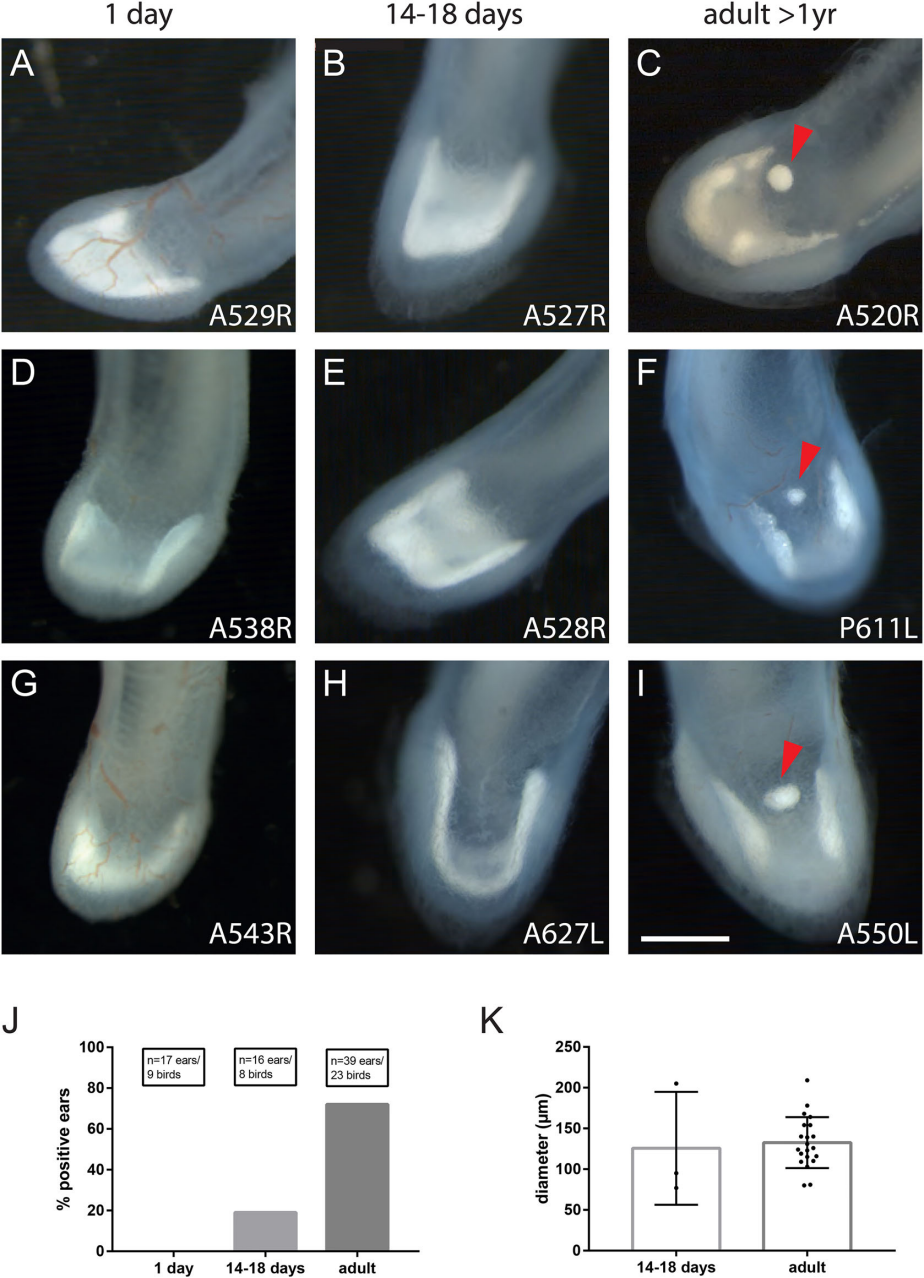
**An otoconial mass (arrowheads) at the dorsal roof of the adult pigeon lagena.** (A-I) Stereomicroscope images of lagenae of freshly hatched (A,D,G), 14-18 days old (B,E,H) and over 1-year-old birds (C,F,I). The number in the lower right indicates the animal number and whether the left (L) or right (R) ear is depicted. (J) The new structure was not observed in 1-day-old birds (*n*=17 ears), rarely in birds aged 14-18 days (19%, *n*=16 ears) and frequently in adult birds (72%, *n*=23 ears). (K) The mean diameter of the structure did not differ between young and adult birds. Bars and error bars represent mean±s.d. Scale bar: 500 µm.

As part of an ongoing study to investigate the anatomical and elemental features of the avian cochlear duct, we observed a small, spherical, otoconial structure at the junction between the lagena and basilar papilla, close to the dorsal epithelium (Fig. 1C,F,I). This structure appeared to be attached to the tegmentum vasculosum and the transitional epithelium. Employing histological methods, we observed this otoconial mass in 72% of cochlear ducts from adult (>1 year of age) pigeons (*n*=39 ears, *n*=23 pigeons, Fig. 1J). The otoconial structure was usually in the dorsal midline of the duct, but occasionally it was located laterally. The analysis of unprocessed paraffin sections revealed that in some, but not all, cases, the structure was encapsulated by a thin, acellular membrane (Fig. 3A,B). The crystal structure of the otoconia appeared to be finer-grained compared to regular lagenar otoconia.

To ascertain when this otoconial structure is formed, we undertook an analysis of birds at hatching. We found no evidence for its existence at this early time point (*n*=17 ears, 9 birds), whereas it was present in 19% of birds aged 14-18 days (*n*=16 ears, 8 birds) (Fig. 1A-J). The average diameter of the structure was 126±57 µm (mean±s.d., *n*=3 ears, 2 birds) in 14-18 day-old birds and 133±31 µm (*n*=21 ears, 15 birds) in adult birds (Fig. 1K). We observed this structure in both left and right ears (left: 81%, *n*=21; right: 61%, *n*=18). The percentage of ears containing this structure was similar in the two different pigeon strains (Vienna cohort: 72%, *n*=32 ears; Nuremberg cohort: 71%, *n*=7 ears).

**Fig. 2.**
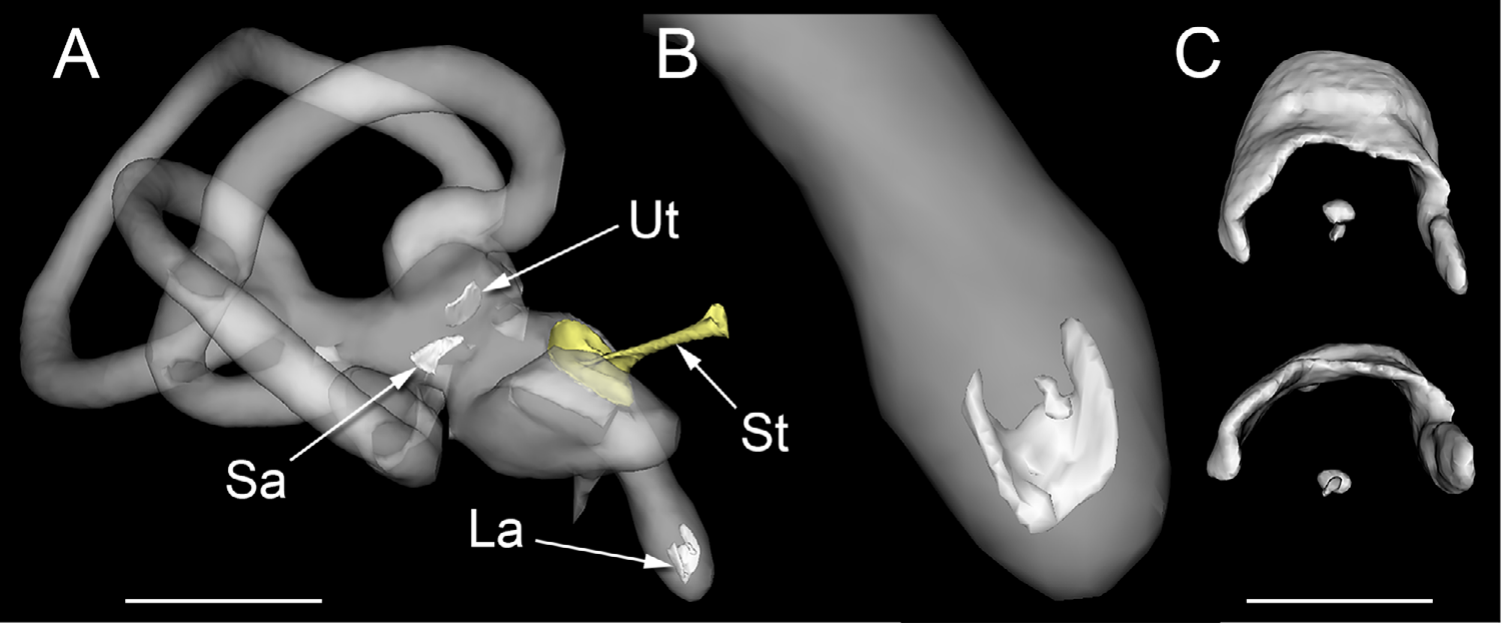
**High resolution µCT reconstructions of the pigeon inner ear (*n*=3** **birds, *n*=6 ears).** (A) Reconstruction of the right pigeon bony labyrinth and stapes (St), showing the otoconial masses within the utricle (Ut), saccule (Sa) and lagena (La). (B) Reconstruction of the pigeon lagena showing the position of the second otoconial aggregate above the main horseshoe-shaped otoconial mass. (C) Reconstructions of two lagenar otoconial masses, illustrating their positions relative to each other. Scale bars: 3 mm (A) and 500 µm (C).

We considered that this otoconial structure might have resulted from the post-mortem displacement of lagenar otoconia during dissection of the cochlear duct. To investigate this possibility, we performed high-resolution computed tomography of the inner ear while it was still embedded within the otic capsule of the temporal bone (*n*=3 birds, *n*=6 ears). This analysis allowed clear visualization of the otoconia within the saccule, utricle and lagena (Fig. 2A). In addition, we observed ectopic otoconia between the lagena and basilar papilla in three of the six ears scanned (one ear from each specimen). These were of similar size and shape to those identified histologically (Fig. 2B-C). These data show that the presence of the otoconial mass is not an artefact associated with cochlear duct dissection.

**Fig. 3.**
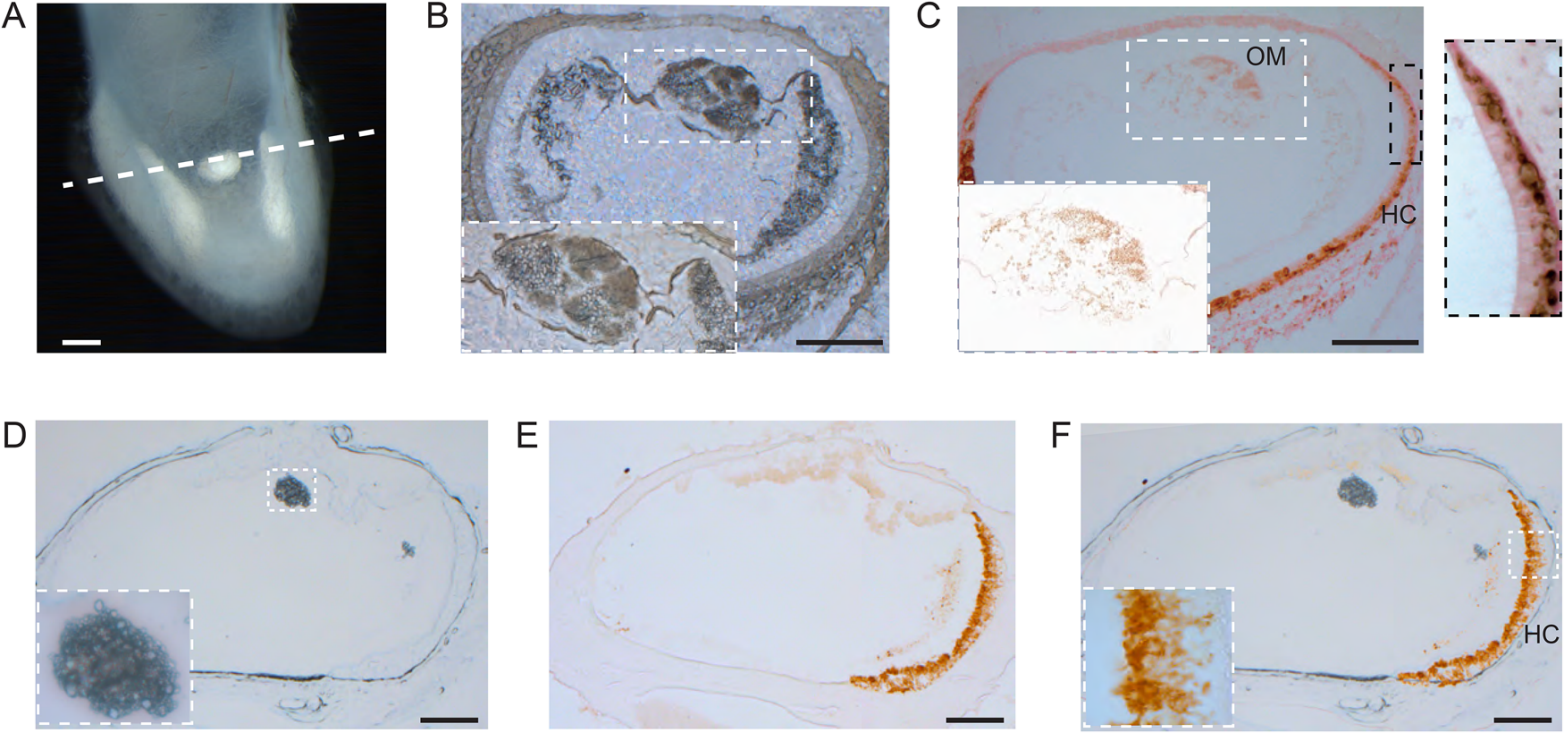
**Histological sections reveal that the otoconia are enclosed by an acellular membrane and are not innervated.** (A) Light micrograph of the pigeon lagena shown in Fig. 1I, with the plane of sectioning indicated by the dashed line. (B) Paraffin section (10 µm) showing the fine crystal structure and membranous margins of the ectopic otoconia. (C) Immunohistological staining with the postmitotic neuronal marker (TuJ1) (*n*=3 birds). Staining at the base of lagenar hair cells (HC, black dashed box) was observed, but no signal was observed in the vicinity of the ectopic otoconial mass (OM, white dashed box). (D) Cryo-section (12 µm) showing the ectopic otoconia, magnified in inset. (E) Immunohistological staining with the hair cell marker otoferlin (*n*=3 birds). The lagenar hair cells (lower right) were stained. (F) Overlay of the unstained and stained section (D,E) illustrating an absence of positive staining in the vicinity of the ectopic otoconia. Scale bars: 100 µm.

We then asked whether this otoconial mass is associated with neuronal structures or hair cells. We undertook histological staining with TuJ1, which labels post-mitotic neurons (Stone and Rubel, 2000) and otoferlin which labels hair cells (Goodyear et al., 2010). We did not observe any TuJ1 positive terminals in the vicinity of the otoconial mass (*n*=3 birds) (see Fig. 3C). Similarly, analysis of histological sections stained for otoferlin failed to identify any hair cells associated with this structure (*n*=3 birds) (see Fig. 3D-F).

**Fig. 4.**
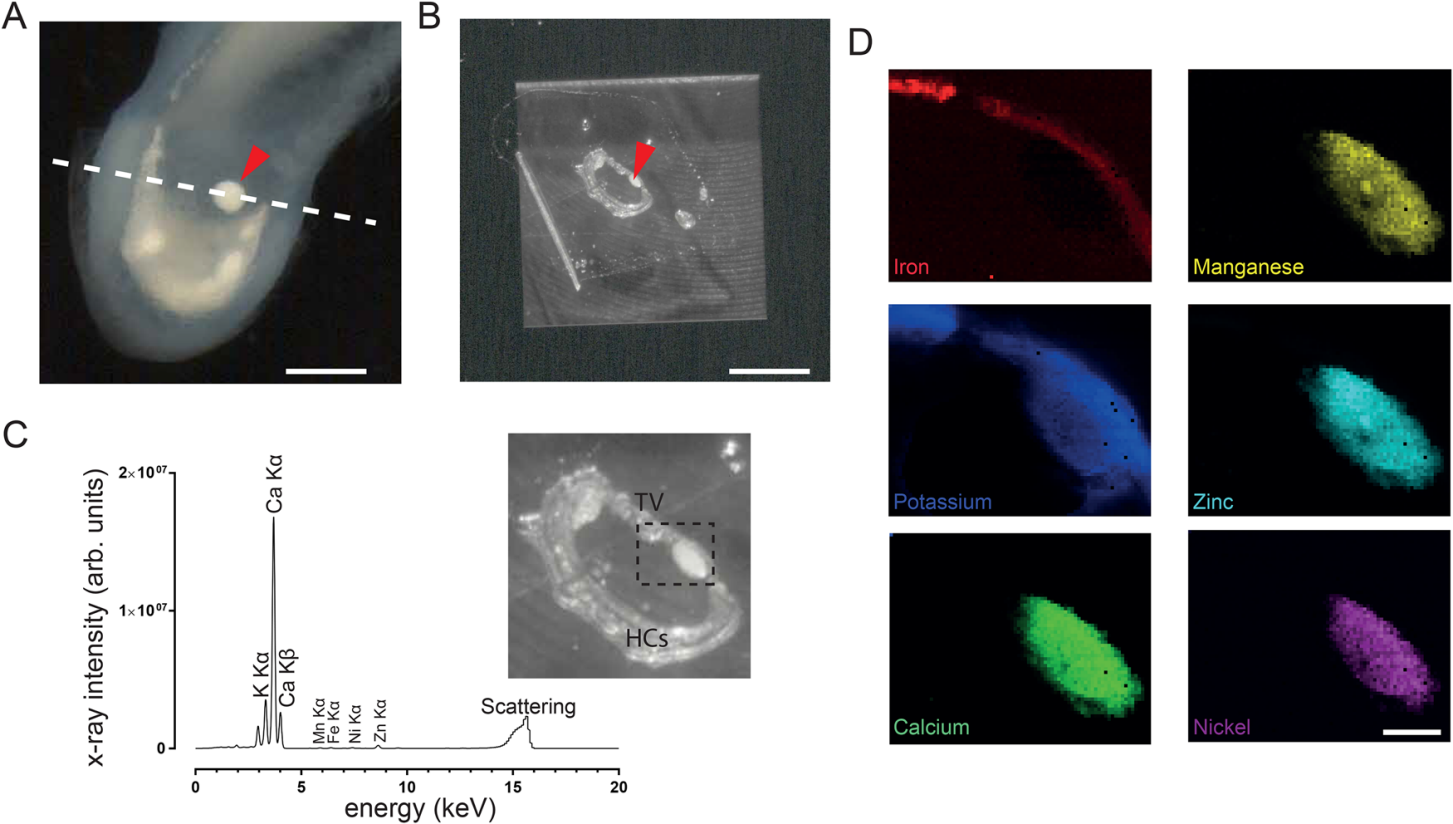
**High resolution elemental analysis using synchrotron x-ray fluorescence microscopy demonstrates high calcium and low trace element concentrations within the additional otoconial mass (bird A520, right ear).** (A) Light micrograph of the pigeon lagena shown in Fig. 1C, with the plane of sectioning indicated by the dashed line. (B) A 60 µm thick cryo-section dried onto a silicon nitride membrane. (C) X-ray fluorescence spectra of the indicated region of interest with the most prominent peaks assigned to elements. Note the close association with the iron-rich tegmentum vasculosum. (D) X-ray fluorescence maps showing the distribution of iron, potassium, calcium, manganese, zinc, and nickel in the region of the otoconial mass. Arrowheads point to otoconial mass. HCs, hair cells; TV, tegmentum vasculosum. Scale bars: 500 µm (A); 1 mm (B) and 50 µm (C).

Finally, we performed synchrotron X-ray fluorescence microscopy to investigate the elemental composition of this curious structure (*n*=2 birds) (Fig. 4A-D). We found that it is dominated by the presence of calcium, represented by two peaks at the calcium K_α_ (3.69 keV) and K_β_ (4.01 keV) edges (Fig. 4C). A further minor peak corresponded to potassium (K_α_ 3.31 keV) and also trace metals were detectable: zinc (K_α_ 8.64 keV), nickel (K_α_ 7.4 keV) and manganese (K_α_ 5.9 keV). The nearby tegmentum vasculosum was characterized by strong potassium signals as well as iron (K_α_ 6.4 keV, Fig. 4D). The additional otoconial mass did not contain detectable amounts of iron. A close association between otoconial mass and tegmentum vasculosum was evident from these images.

## DISCUSSION

**Fig. 5.**
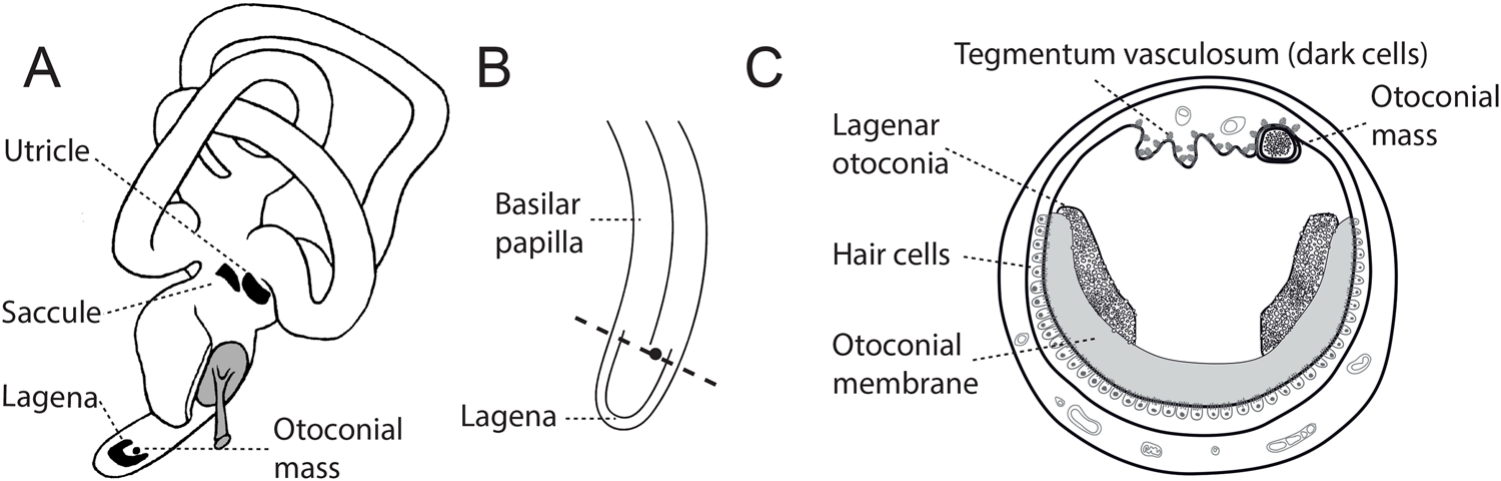
**Model showing the additional otoconial mass in the pigeon inner ear lagena.** (A) Diagram of the pigeon inner ear, indicating the position of the three otoconia-containing organs: saccule, utricle and lagena. The small dot in the lagena shows the position of the newly-discovered otoconial mass. Sketch based on a reconstruction of the bony labyrinth from µCT scans of a pigeon right ear. (B) Diagram indicating the transitional area between the pigeon basilar papilla and lagena where the additional otoconial mass is located. (C) Diagram of a lagena in the plane indicated by the dashed line in B. The close relationship of the additional otoconial mass to the dorsal tegmentum vasculosum is depicted.

We report the discovery of a calcium-rich structure located at the junction between the lagena and basilar papilla in the pigeon inner ear. It superficially resembles other otoconial masses of the avian inner ear, but is distinguished by its location and finer crystalline appearance. It is usually enclosed by a thin, acellular membrane and is suspended from the dorsal tegmentum vasculosum (Fig. 5). The tegmentum vasculosum, the avian equivalent of the mammalian stria vascularis, lines the dorsal part of the saccule, basilar papilla and lagena and is associated with endolymph production. This process is performed by dark cells, a specialized set of cells with extensively enlarged luminal surfaces (Ishiyama et al., 1970).

The origin and function of the additional otoconial mass is unknown. It could be: (a) involved in sensory perception, acting either together with or separate from the lagenar macula; (b) an accumulation of displaced lagenar otoconia; or (c) an ectopic, *de novo* formation of otoconia. A sensory function would appear unlikely given that we do not observe any association with neuronal structures or hair cells. It seems improbable that the structures are displaced otoconia resulting from age-related factors or experimental artefacts. The lagenar otoconia are notably different, being larger in size with a distinct shape, whereas the otoconia we describe are smaller and more densely packed. Moreover, the structures we report were often encapsulated by a membrane again suggesting that they are not the product of displacement.

Could the structures represent *de novo* formations? We observed the otoconial mass to be immediately adjacent to the dark cells of the tegmentum vasculosum. Dark cells, the avian equivalent of the marginal cells of the mammalian stria vascularis (Wangemann, 1995), are responsible for maintenance of the endolymph ionic composition which is essential for inner ear function. Dark cell populations in the vertebrate utricle and saccule have also been implicated in the turnover of otoconia (Harada and Sugimoto, 1977; Lim, 1973; Preston et al., 1975). In mice, the Ca^2+^-ATPase PMCA2, which is expressed in dark cells, is required for otoconia formation, and might therefore be a key transporter used in the export of calcium ions into the endolymphatic space (Kozel et al., 1998; Lundberg et al., 2006). Mammalian dark cells express carbonic anhydrase (Lim et al., 1983). This enzyme is used in the production of HCO_3_^−^, which is believed to be involved in the formation of the calcium carbonate otoconia. Dark cells also express otoconin-90 (Oc90), a matrix protein required for otoconial seeding (Lundberg et al., 2006). The presence of Oc90 protein precipitate has been related to transient, ectopic otoconial formation in the endolymphatic sac of developing mice (Ignatova et al., 2004). Accordingly, it has been suggested that vestibular dark cells form minute calcium crystal seeds in the endolymph (Lundberg et al., 2006). Dysregulation of this process or enhanced crystallization at these seeds (e.g. due to local Ca^2+^ hypersaturation of the endolymph) could lead to the formation of additional otoconia.

Why would such additional otoconia form so frequently in the pigeon inner ear? The trace metals zinc and manganese are critically involved in otoconial formation (reviewed in Erway et al., 1986; Fermin et al., 1998). Carbonic anhydrase contains zinc, while manganese is essential as a cofactor of enzymes needed for the synthesis of a functional otoconial membrane, required for crystal formation. Rats and mice raised on a zinc or manganese deficient diet do not form otoconia (Erway et al., 1986; Huygen et al., 1986). Interestingly, the phenotype can be rescued, but manganese over-supplementation leads to extra-macular otoconia formation (Erway et al., 1986). High manganese and zinc concentrations have been reported in the avian lagena (Harada et al., 2001), conditions that could facilitate *de novo* formation of ectopic otoconia. This is supported by the detection of zinc and manganese within the otoconial mass in our elemental analysis.

It has been proposed that the pigeon lagena contains receptors that allow birds to detect the geomagnetic field (Harada, 2008; Harada et al., 2001; Wu and Dickman, 2011). The primary magnetoreceptors have not been described (Nordmann et al., 2017), but it is conceivable that they consist of biogenic iron oxides such as magnetite (Fe_3_O_4_) (Winklhofer and Kirschvink, 2010). While we detected zinc and manganese in the otoconial mass, the absence of iron and the fact that the structure is not associated with sensory hair cells suggests that it is unlikely to be associated with a magnetic sense.

In summary, we report the discovery of an ectopic otoconial mass in the lagena of the majority of adult pigeons. We suggest that local ionic conditions in the pigeon lagena favour formation of calcium carbonate crystals, which aggregate close to the lagenar roof. Whether the aggregates affect lagenar function is unknown. The frequent occurrence of these ectopic otoconia could make them a convenient model for the study of processes involved in otoconial formation during aging.

## MATERIALS AND METHODS

### Tissue preparation

Pigeons (*Columba livia domestica*) from two different cohorts (Vienna and Nuremberg) were utilised in this study. The animals were euthanized and intracardially perfused with 40°C 0.1 M phosphate-buffered saline (PBS) supplemented with 20 U/l heparin, followed by ice-cold 4% phosphate-buffered paraformaldehyde (PFA). The inner ears were removed from the skull, and the oval window and the superior semicircular canal opened to facilitate the penetration of the fixatives. The tissue was postfixed overnight in 2%PFA/2.5% glutaraldehyde (GA) or 4% PFA at 4°C. In some instances ears were fixed overnight without prior intracardial perfusion. Following postfixation, the samples were washed in PBS for 1 h, and the lagenae were exposed by dissection under light microscopy. Images of the lagena were taken at 20× and 40× magnification through a Leica MZ 16 FA stereomicroscope. The dimensions of the observed structures were measured from the resulting images using ImageJ (v.1.51r, NIH). The tissue was then embedded in paraffin or frozen section medium (Neg-50; Richard-Allan Scientific, Kalamazoo, MI, USA) and sectioned coronally (10 or 12 µm).

### Immunohistochemistry

For staining of nerve terminals, we used paraffin sections (10 µm). Primary antibodies against class III beta tubulin (TuJ1 MMS-435P, Lot #D13AF00117, Covance, 1:500; Lee et al., 1990) were incubated in 2.5% normal horse serum (NHS) in PBS overnight at room temperature. After three washes in PBS the secondary antibodies (MP-7402, Vector Laboratories, Burlingame, CA, USA) were incubated for 1 h at room temperature. Following an additional three PBS washes the staining was visualized with the chromogen 3,3′-diaminobenzidine (SK-4105, Vector Laboratories). Nuclear Fast Red (C.I. 60760; Carl Roth, Karlsruhe, Germany) served as a counterstain. Hair cells were stained on cryo-sections (12 µm). After a heat-mediated antigen retrieval (H-3301, Vector Laboratories) the sections were washed three times in PBS and primary antibodies against otoferlin (Sc-50159, C-15, Lot #A2930; Santa Cruz, 1:1000) were incubated in 2% milk, 0.1% Triton in PBS overnight at room temperature (Lauwers et al., 2013). After three washes in PBS the secondary antibodies (MP-7405, Vector Laboratories) were incubated for 1 h at room temperature. Staining was than visualized with DAB as described above.

### Elemental analysis

Synchrotron x-ray fluorescence microscopy (XFM) was performed on sections of two lagenae at beamline B16 of the Diamond Light Source, Oxford, UK. The tissue was dissected as described above. Following postfixation and washing, the samples were immersed in OCT mounting medium (Neg-50, Richard-Allan Scientific) and rapidly frozen in liquid nitrogen-cooled isopentane. Using a ceramic-coated blade (DuraEdge High Profile BLM00103P; American MasterTech, Lodi, CA, USA), 60 µm cryo-sections were prepared using a cryostat (−20°C) and thawed onto 7.5 mm diameter silicon nitride frames (membrane thickness 1 µm; Silson, Southam, UK). The samples were air-dried and kept at room temperature until analysis. XFM-maps were collected at 18 keV with a beam size of 0.5 µm and a step size of 5 µm. We used a 4-element Vortex detector and dwell times of 2-8 s per pixel. Elemental fits and maps were performed in PyMca (Solé et al., 2007). X-ray absorption edges were taken from the online version of the Kaye & Laby Tables of Physical & Chemical Constants (http://www.kayelaby.npl.co.uk/atomic_and_nuclear_physics/4_2/4_2_1.html).

### CT scans

Pigeons (Vienna cohort) were perfused as described, the ear regions grossly dissected and postfixed in PFA overnight. To enhance contrast, one ear specimen from each pigeon was incubated in Lugol solution (L6146; Sigma-Aldrich) for several days before scanning. The samples were separately mounted in a Nikon XT H 225 CT-scanner at the Cambridge Biotomography Centre, Cambridge, UK. Two 1000 ms exposure images, made using settings of 125 kV and 120 µA, were averaged at each of 1080 projection angles. Reconstruction software included CT Agent XT 3.1.9 and CT Pro 3D XT 3.1.9 (Nikon Metrology, 2004–2013). Cubic voxel side-lengths were from 10-14 µm. The 16-bit tomograms were converted to 8-bit tiff or jpg files in Photoshop CS 8.0 (Adobe Systems Inc., 2003). 3D reconstructions of the stapes, bony labyrinth and otoliths were made from the jpg files using WinSurf 4.0 (E. Neufeld, 2001), in a procedure requiring manual identification of boundaries from the tomograms. More detailed reconstructions of the lagenar otoliths and any associated calcified structures were made from the tiff files using MicroView 2.5.0 (Parallax Innovations Inc., 2017).

## Supplementary Material

First Person interview
