# Supplementary material for: Ectopic otoconial formation in the lagena of the pigeon inner ear
Source: Biol Open. 2018 Jun 26;7(8):bio034462. doi: 10.1242/bio.034462 (PMC6124575; doi:10.1242/bio.034462)
Supplement: First Person interview [file biolopen-7-034462-s1.pdf]

© 2018. Published by The Company of Biologists Ltd | Biology Open (2018): doi:10.1242/bio.034462: Supplementary information

FIRST PERSON

First person – Pascal Malkemper

First Person is a series of interviews with the first authors of a selection of papers published in Biology Open, helping early-career researchers promote themselves alongside their papers. Pascal Malkemper is first author on 'Ectopic otoconial formation in the lagena of the pigeon inner ear', published in BiO. Pascal is a postdoc in the lab of David Keays at the Research Institute of Molecular Pathology, Vienna, Austria, investigating the cellular basis of the magnetic sense in vertebrates.

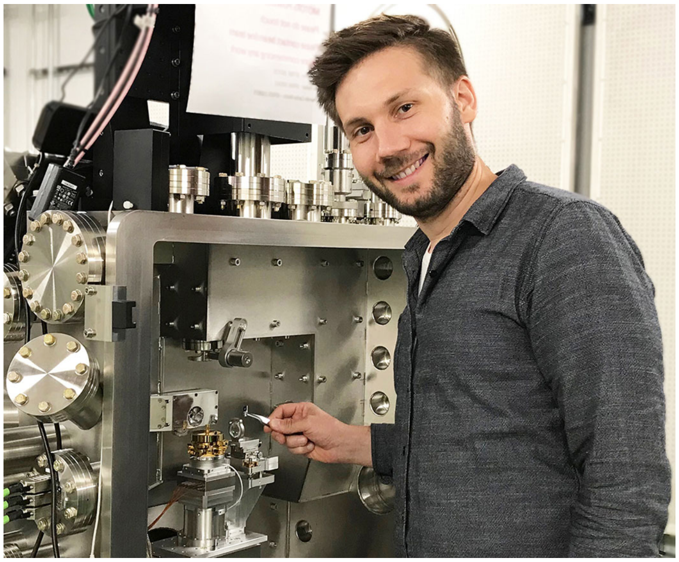

Pascal Malkemper mounting a pigeon inner ear sample at the I14 hard X-ray nanoprobe beamline of the Diamond synchrotron in Oxford.

What is your scientific background and the general focus of your lab?

I studied biology and neuroscience. In my PhD, supervised by Prof. Hynek Burda, I tackled the relationship between the morphology of a sensory organ and its function using the red fox as a model system. I spent several months doing field work in the Czech Republic and combined the results with lab work done in Germany. My current research focusses on the magnetic sense, an exciting field because even after half a century of research, the receptors that allow animals to orient by the geomagnetic field have not been described. Using mole-rats and pigeons as model species, I employ behavioral experiments and cutting-edge neurohistology to find the primary receptor cells.

How would you explain the main findings of your paper to non-scientific family and friends?

We studied the lagena, a part of the inner ear of birds that humans do not have. The function of this organ is not well known, but it has been suggested to play a role in the magnetic sense. Searching for iron that could constitute a magnetoreceptor in the pigeon lagena, we noticed a peculiar round aggregate of calcium carbonate at a very specific location that had not been mentioned in the literature. It is best described as a ball of calcium crystals hanging from the ceiling of the lagena. We hypothesized that it might have a sensory function but studying it in detail did not provide evidence for that. Instead, we concluded that what we observed was likely to be a malformation that forms when pigeons age.

What are the potential implications of these results for your field of research?

The structure we discovered does not seem to have a sensory function, however, it may be of clinical relevance. Humans do not have a lagena, but the calcium crystals we studied are a crucial component of our sense of balance. Defects in the inner ear that affect balance are still amongst the most common age-related diseases. The structure we describe may serve as a useful model to gain insights into the processes that lead to vestibular dysfunction.

"In my view, such accidental discoveries highlight the value of basic, curiosity-driven research."

What has surprised you the most while conducting your research?

It was astonishing that one can still discover major new structures in sensory organs, even in well-studied systems like the pigeon inner ear. These rare moments when you set eyes on something that no other human has seen before are both exciting and motivating. In my view, such accidental discoveries highlight the value of basic, curiosity-driven research.

What, in your opinion, are some of the greatest achievements in your field and how has this influenced your research?

In the field of magnetoreception, the value of the seminal studies by the Wiltschkos that unambiguously showed that birds use the Earth's magnetic field for orientation cannot be overestimated. Nowadays, however, the most important studies to me are those that clearly highlight the limits of applied methods and critically assess what we really know and learned from the findings. Data on the magnetic sense of animals is notoriously hard to collect and sometimes hard-earned findings are over-interpreted. Critical studies, such as one that revived the question of whether birds really have magnetoreceptors in their upper beak, are vital for the field.

What changes do you think could improve the professional lives of early-career scientists?

There is a need for reliable long-term prospects in the form of more tenure-track positions. The decision on whether a young graduate is going to stay in academia science should happen at an earlier stage in their career to ensure that Uber is not inundated with highly educated applicants. Career development plans that inform graduates about alternative paths before starting a PhD would be beneficial, reducing the bottleneck between postdoc and permanent positions.

Pascal Malkemper's contact details: Research Institute of Molecular Pathology, Campus Vienna Biocenter 1, Vienna 1030, Austria.

E-mail: erich.malkemper@imp.ac.at

What's next for you?

I will stay on the hunt for vertebrate magnetoreceptors, making use of the great variety of techniques available in the Keays lab. Simultaneously, I am writing grant proposals to establish my own research group to study the role of the magnetic sense in the spatial orientation of mammals.

Reference

Malkemper, E. P., Mason, M. J., Kagerbauer, D., Nimpf, S. and Keays, D. A. (2018). Ectopic otoconial formation in the lagena of the pigeon inner ear. *Biol. Open* **7**: bio034462.
